# Corporate ESG and total factor productivity: Will the fulfillment of social responsibility sacrifice productivity?

**DOI:** 10.1371/journal.pone.0301701

**Published:** 2024-04-25

**Authors:** Jian Chen

**Affiliations:** School of Finance, Southwestern University of Finance and Economics, Chengdu, China; University of Cagliari: Universita degli Studi Di Cagliari, ITALY

## Abstract

With the increasing prominence of climate and energy issues, enterprises, as the micro-subjects of economic activities, need to pay attention to environmental responsibility to promote sustainable and high-quality economic development. However, one of the crucial controversies is whether enterprises will sacrifice efficiency to fulfill their environmental responsibilities. To try our best to answer the controversy, this paper explores the impact of ESG on total factor productivity and its mechanism. The research conclusion shows that Chinese enterprises fulfilling ESG responsibilities can improve staff efficiency, reduce financing costs, ease financing constraints, and increase innovation investment, thus effectively improving total factor productivity. Compared to non-state-owned enterprises, this effect is more significant in state-owned enterprises. In addition, the promotion of ESG construction on the total factor productivity of enterprises also presents specific acceleration characteristics. This shows that in the socialist market economy environment, there is an obvious "social responsibility dividend" in the implementation of the ESG concept by Chinese enterprises, which is helpful to enhance their long-term value and realize a win-win of social value and commercial value. The conclusions of this study help deal correctly with the relationship between business value and social value of enterprises and provide inspiration for promoting healthy and sustainable economic development.

## 1. Introduction

In recent years, sustainable development issues such as climate change, energy shortage, and the gap between the rich and the poor have attracted much attention. As the micro subject of economic development, the rapid development of enterprises has effectively promoted the promotion of the macroeconomic level. However, while pursuing commercial value, some enterprises still have short-sighted behaviors, such as environmental pollution, according to statistics from the World Health Organization and the International Labour Organization, the environmental costs caused by the global economy development each year are approximately $4.7 trillion, which aggravate the problem of sustainable development in economic society. The party’s Report to the 20th CPC National Congress pointed out that promoting green and low-carbon economic and social development is the key to achieving high-quality development. How to promote green development of enterprises and achieve a win-win situation between social and commercial value is an important research topic for high-quality economic development.

ESG (Environment, Social and Governance) is an integration and progress of concepts, such as corporate environment, social responsibility, and sustainable development, and has gradually become an indispensable part of high-quality development for enterprises. However, there are still some critical issues to be resolved in the ESG practice of enterprises: will there be conflicts between enterprises’ ESG responsibilities and their business value objectives? What is the relationship between ESG and its economic efficiency? Therefore, this paper discusses how ESG affects its total factor productivity, aiming at clarifying the relationship between corporate environmental responsibility and its economic efficiency, which is of great significance in promoting the win-win of corporate commercial value and social value and promoting the high-quality development of enterprises.

The relationship between corporate social value and commercial value is still controversial in the literature. One view is that enterprises can help improve the business value of enterprises while fulfilling their social responsibilities. Specifically, the good ESG performance of the enterprise contributes to the creation of intangible assets such as human capital and corporate reputation [1, 2], Reducing Financing Costs [3, 4], Improving Corporate Performance [5], and shaping corporate image [6, 7]. When examining the impact of green finance policies on corporate social responsibility, Huang et al. concluded that corporate social responsibility can promote economic benefits through green innovation, improving total factor productivity, and expanding market share [8]. In addition, they also confirmed that green development models and digital finance inclusion are important driving factors for developing sustainable economies [9–13]. Another point of view is that an enterprise’s investment in ESG will also damage its profitability and financial performance. Specifically, ESG construction may be used by managers to obtain private benefits at the expense of shareholders [14, 15]. Managers may collude with stakeholders other than shareholders to protect them from internal disciplinary mechanisms [16]. Enterprise ESG construction may also lead to over-investment, increase costs, and erode profits [17]. These studies generally help understand the relationship between ESG and corporate value. However, there is no clear answer to the relationship between ESG and the quality of enterprise development (production efficiency).

To make up for the deficiencies of the existing research, this paper examines the impact of ESG on total factor productivity and its mechanism. By selecting A-share listed companies from 2009 to 2021 as the research object, this paper uses the LP method to estimate the total factor productivity level [18, 19], constructs the relationship model between ESG and total factor productivity for empirical analysis, and further discusses the potential mechanism from the aspects of staff efficiency, capital constraint, and innovation investment. To ensure the robustness of the conclusions, we control the potential endogenous problems utilizing the instrumental variable method, and further test using replacing different assignments, different measures, and adding time lag term. The study found that ESG construction of Chinese enterprises did have a significant positive impact on total factor productivity, especially for state-owned enterprises. In addition, the promotion of ESG construction on enterprise total factor productivity also shows specific acceleration characteristics. The mechanism behind it is that ESG construction can effectively improve staff efficiency, reduce financing costs, ease financing constraints, and increase investment in innovation to achieve production efficiency. This indicates an obvious "social responsibility dividend" in the practice of the ESG concept in Chinese enterprises, which aligns with the new development concept advocated by the CPC Central Committee. It indicates that it is urgent to improve the ESG level of enterprises, promote the improvement of total factor productivity, and promote the win-win of social value and commercial value to achieve high-quality economic development.

The main contributions of this article are as follows: Firstly, existing literature mostly focuses on exploring the impact of ESG on enterprise commercial value, and there is no clear answer to the relationship between ESG and the quality of enterprise development (production efficiency). This article clarifies the impact and mechanism of corporate ESG on total factor productivity through empirical analysis, filling the research gap mentioned above and helping to better achieve a win-win situation between corporate social value and commercial value. Secondly, this article finds that employee efficiency, capital constraints, and innovation investment are the mechanisms by which the level of enterprise ESG affects total factor productivity. This provides a deeper understanding for better promoting enterprise ESG to promote total factor productivity and promote high-quality development. Thirdly, this article proposes the existence of a "social responsibility dividend" in China’s market economy, which is in line with the current advocated new development concept. Chinese enterprises need to seize the opportunity of social responsibility dividends, actively fulfill ESG responsibilities, and achieve the co creation of commercial value and social value, thereby providing suggestions and inspiration for policy makers.

## 2. Theoretical analysis and research hypothesis

### 2.1 ESG and total factor productivity

Total factor productivity refers to the "surplus" in total output that cannot be explained by factor inputs. It refers to economic growth that can be explained by technological progress, resource allocation efficiency improvement, organizational and institutional innovation, economies of scale, and other factors beyond capital and labor inputs. Total factor productivity is an important reference indicator for measuring production efficiency and technological progress, and an important standard for measuring the quality of enterprise development [18, 19]. However, it is not always consistent with an enterprise’s short-term profit and long-term value, and enterprise operators often have to make a choice between the two. For example, for product innovation, due to its large upfront investment and high input-output uncertainty, the Innovation investment may affect the current financial performance of the enterprise. However, it is beneficial to the growth of the enterprise. Due to the principal-agent problem between the shareholders and the operators, the operators are likely to avoid such investment due to the consideration of current profit performance appraisal [20, 21], which may hinder the long-term development of the enterprise and prevent the win-win of commercial value and social value.

Advancing Enterprise ESG can help solve this problem. However, the relationship between ESG and total factor productivity is still controversial. One view is that the development of ESG is contrary to the improvement of total factor productivity. On the one hand, enterprises exist to develop productivity at the expense of social interests. Compared with investing a large amount of resources in technological innovation and market development to improve total factor productivity, chief executive officers often achieve this by reducing the consumer surplus and the producer surplus of downstream manufacturers, because this approach does not require a large amount of expenses and time, and does not need to bear too much risk. On the other hand, ESG performance exists to improve enterprise productivity. Li and Liu (21) believe enterprises may "pay too much attention to social value investment." To improve their social image, enterprise managers may invest too many resources in image packaging and staff relationship maintenance, thus reducing the investment in innovation and research, resulting in a decline in total factor productivity. Therefore, the investment in ESG construction of enterprises is contrary to the improvement of total factor productivity.

Another view is that enterprises can promote total factor productivity through ESG construction to achieve a win-win situation between social value and commercial value. Jones, Willness (1), Porter and Kramer (7) found that enterprises can improve their corporate image through ESG construction, attract talented job seekers, and thus improve their productivity. El Ghoul, Guedhami (3), Goss and Roberts (4) believe that enterprises can ease financing constraints and reduce financing costs through ESG construction, and invest more and cheaper funds in research and development of core technologies and products, thus improving enterprise productivity. Therefore, ESG construction of enterprises can improve total factor productivity.

What is the relationship between ESG and total factor productivity? Will the investment in ESG construction cost the economic efficiency of the enterprise? In response to these problems, this paper believes that under the increasingly tricky social responsibility challenges brought by sustainable development, those enterprises that value the ESG concept will establish specific advantages, improve total factor productivity, and achieve a win-win situation of commercial value and social value. The root causes are as follows: First, good ESG performance is conducive to sending positive signals to the outside world to actively protect the rights and interests of stakeholders such as employees, which not only attracts talented job seekers to join, but also enhances the initiative and enthusiasm of employees, improves employee efficiency, and thus promotes total factor productivity; Second, practicing ESG concept will help to send positive signals to financial intermediaries such as banks and capital markets, ease information asymmetry, improve investors’ mastery of non-financial information of enterprises, and provide financing support for technological innovation and equipment upgrading of enterprises; Third, ESG construction helps to break through the dilemma brought by the principal-agent problem, avoid the selfish and short-sighted behavior of managers, improve the tolerance of stakeholders for high-uncertainty projects such as product innovation, provide a relaxed environment, and promote the innovation investment of enterprises. Based on the above analysis, we put forward the following hypothesis:

**Hypothesis 1**: Enterprises can improve total factor productivity and long-term value by practicing the ESG concept, and realize a win-win of social value and commercial value.

### 2.2 ESG performance, staff efficiency, and total factor productivity

The labor force is the core factor of production, and an enterprise’s production and operation activities need the support of employees. Improving the efficiency of employees is of great significance in promoting total factor productivity. Due to the business philosophy of "maximizing the interests of shareholders," employees’ interests are often not fully valued and protected, and employees’ sense of ownership and sense of belonging are not strong enough, which weakens employees’ initiative and enthusiasm. At the same time, since employees cannot share the fruits of enterprise development, individual talents are often not entirely played. In addition to the complex management structure, staff communication and cooperation efficiency are low. Therefore, how to motivate the staff’s subjective initiative and improve staff efficiency has become the critical issue in enhancing the total factor productivity of the enterprise.

Implementing the ESG concept provides a feasible solution to this problem. First, the ESG concept reshaped the concept of enterprise development, extending from "maximizing shareholders’ interests" to stakeholders, especially employees. Implementing the ESG concept has sent a positive signal to the outside world to protect the rights and interests of stakeholders such as employees, attracted talented job seekers, improved the centripetal force and cohesion of the enterprise, and improved the efficiency of production collaboration among employees, thus improving the total factor productivity [1]. Secondly, ESG construction stimulates employees’ subjective initiative. Establishing an incentive compensation system and improving staff welfare levels are all essential links in ESG construction. The employee stock ownership plan and other incentives help promote the interests of employees, improving the staff’s subjective initiative [20]. Therefore, this paper puts forward the following hypothesis:

**Hypothesis 2**: Enterprises can improve total factor productivity by implementing ESG philosophy to improve staff efficiency.

### 2.3 ESG performance, capital constraints, and total factor productivity

The development of enterprise production also needs capital support. The capital constraint has been one of the significant obstacles for enterprises to improve productivity. Li and Liu (21) pointed out that enterprises with loan difficulties lack the funds to support equipment upgrading and technological improvement, resulting in the loss of total factor productivity. Yu, Wu (22) also found that the increase in financing cost hindered enterprises’ technological innovation and product equipment renewal, which hurt the total factor productivity of enterprises. El Ghoul, Guedhami (3), Goss and Roberts (4) believe that reducing financing costs and easing financing constraints can promote technological innovation and equipment upgrading of enterprises, thus improving the total factor productivity of enterprises.

However, due to the asymmetric information between the lenders and the borrowers, it is challenging for banks and other financial institutions to identify competent borrowers from a large number of loan contracts. Therefore, even though some enterprises have good business prospects and are willing to bear higher capital costs, they cannot obtain loans [22]. A large number of small and medium-sized enterprises, especially private enterprises, have difficulty in obtaining financing from outside due to their small scale and lack of government endorsement, which limits their ability to expand production, improve technology, and product innovation [4]. In addition, companies also face financing costs from higher interest rates [3]. Therefore, how to ease the capital constraint problem has become one of the significant problems that must be solved in developing enterprise productivity.

The practice of the ESG concept and the establishment of ESG advantages provide effective means to solve this problem. On the one hand, the good ESG performance of the enterprise helps to reduce the financing cost, thus enhancing the willingness of the enterprise to upgrade the equipment and improve the technology, promoting the total factor productivity [3, 4]. Specifically, (1) from the perspective of indirect financing, based on the information asymmetry theory, ESG performance, and information disclosure of enterprises can effectively reduce the information asymmetry, improve financial institutions such as banks or investors’ mastery of the information of enterprises, improve the accuracy of their investment decisions and reduce their risk premium requirements, thus easing the financing difficulties of enterprises and reducing the financing costs [4]. (2) from the point of view of direct financing, based on the signaling theory, the ESG performance of enterprises will send positive signals to the market, which enhances the confidence of market investors, improves the investment willingness, and alleviates its financing constraints [22]. (3) ESG performance directly affects investors’ investment considerations. Sustainable development has become the consensus of the whole society, and ESG is the index to evaluate corporate social responsibility, so investors often choose to invest in ESG’s better-performing enterprises [23].

Based on the above analysis, we put forward the following hypothesis:

**Hypothesis 3**: Enterprises can improve total factor productivity by implementing the ESG concept to reduce financing costs and ease financing constraints.

### 2.4 ESG performance, innovation investment, and total factor productivity

Enterprise Innovation investment is also a critical factor in determining total factor productivity. Generally, the lack of investment in enterprise innovation is mainly due to the principal-agent problem. Although product innovation is the key for an enterprise to obtain a competitive advantage and excess profits in the industry, product innovation has the characteristics of a large upfront investment and a long cycle. It has excellent uncertainty, which will affect the current profits of the enterprise and worsen the financial performance [14]. At the same time, because the owners often conduct performance appraisals on the operators through the short-term profits of the enterprise, the operators are more inclined to projects with short-term and stable cash flow due to the consideration of their interests and reputation. They are unwilling to invest in highly uncertain projects like product innovation [21]. At the same time, due to asymmetric information, external stakeholders (mainly financial institutions and investors) lack adequate supervision over the operation and management of enterprises and tend to invest in projects with short investment cycles and stable cash flows [24].

Implementing the ESG concept can alleviate the principal-agent problem between owners and operators. On the one hand, the implementation of the ESG concept has sent a positive signal to the outside world that enterprises should fulfill their social responsibilities, indicating that business operators have extended their loyalty to owners to all stakeholders, thus improving the trust of all stakeholders in the enterprise, providing a relaxed environment for enterprise innovation investment, and helping to improve the willingness of enterprise innovation investment [25]. On the other hand, compared with short-term profits, the performance appraisal incorporating the ESG concept can give full play to the role of "baton" to urge business operators to no longer limit themselves to short-term interests and pay more attention to the long-term development of enterprises, thus increasing Innovation investment and promoting total factor productivity [26].

Based on the above analysis, we put forward the following hypothesis:

**Hypothesis 4**: Enterprises can improve total factor productivity by implementing the ESG concept to promote Innovation investment level.

## 3. Variables, models, and data

### 3.1 Measure of variables

#### 3.1.1 Total factor productivity of enterprises: LP method

To overcome simultaneity bias and selectivity bias in estimating total factor productivity, Olley and Pakes [27] proposed the OP method. The method solves the simultaneity bias by using the current investment of the enterprise as the proxy variable of the total factor productivity shock. It solves the sample selectivity bias by constructing an enterprise survival probability model to estimate the entry and exit decisions of the enterprise. However, the OP method requires the actual investment of the enterprise to be greater than zero when calculating the enterprise TFP, which will result in the loss of many data samples. Given this, Levinsohn and Petrin [19] improved the OP method and proposed the LP method. The LP approach does not use investment as an agent variable but uses intermediate inputs as an agent variable for total factor productivity shocks. In addition, Ackerberg, Caves [28] introduce labor input into the intermediate input function and propose the ACF method. However, in general, the assumptions of OP and ACF are too strict. OP requires the actual investment of the enterprise to be greater than zero. The ACF method assumes that the labor adjustment cost is high and requires labor as a free variable. Therefore, this paper uses the LP method to estimate the total factor productivity (*TFP*) of listed companies and uses the OP or ACF method as a robustness test.

Based on the method for calculating enterprise TFP in Levinsohn and Petrin [19], the estimation model of the LP method is as follows:

$$lnY_{it}=\eta_{0}+\eta_{k}K_{it}+\eta_{l}L_{it}+\eta_{a}age_{it}+\eta_{s}state_{it}+\sum \eta_{m}year_{m}+\sum \eta_{n}area_{n}+\sum \eta_{\gamma }ind_{\gamma }+\varepsilon_{it}$$ (1)

Among them, *Y* represents the operating income of the enterprise, *K* represents the capital measured by the net fixed assets of the enterprise, *L* represents the average annual number of employees in the enterprise, *Age* represents the age of the enterprise, and state, year, area and ind respectively represent whether they are virtual variables of state-owned enterprises, year, region and industry. In addition, the intermediate cost of goods purchased and services received is selected as the proxy variable of total factor productivity. Concerning the previous literature above, since Eq (1) already controls the enterprise attribute, year, region, industry, etc., it is no longer necessary to consider industry and year differences when calculating total factor productivity. When Eq (1) is estimated, the logarithmic value of the obtained residual is total factor productivity (*TFP*) as the explained variable.

#### 3.1.2 Enterprise ESG performance

Compared with several commonly used mainstream rating databases such as FTSE Russell ESG rating and Wind ESG rating, Huazheng ESG rating data covers more listed companies (the data of China listed companies rated by FTSE Russell ESG are missing) and has a more extended period (Wind ESG rating does not have the data before 2017). Therefore, this paper selects Huazheng ESG rating data as the core indicator to measure the ESG performance of enterprises and as the explanatory variable. The ESG rating of Huazheng has nine significant grades, with the grades from good to bad in order: AAA, AA, A, BBB, BB, B, CCC, CC, and C. This study assigns nine grades from C to AAA with 1 to 9 points (as shown in Table 1 below).

**Table 1:** Huazheng ESG rating assignment rules.

ESG rating	AAA	AA	A	BBB	BB	B	CCC	CC	C
Assignment	9	8	7	6	5	4	3	2	1

#### 3.1.3 Other control variables

In order to control the influencing factors of total factor productivity (*TFP*) of enterprises as much as possible, the following variables are controlled with reference to the practices of Xu and Zhao [26], Chen, Ma [29]: (1) *CORPSCALE*, which classifies the samples of listed companies into four categories, namely, micro, small, medium and large, with values ranging from 1 to 4 according to the industry classification standard of national economy and relevant regulations; (2) *NATURE*, if the company is a central enterprise or a local state-owned enterprise, the *NATURE* of the company is 1, the other is 0; (3) *INDUSTRY*, which is coded according to the latest Industry Classification Guide for Listed Companies issued by the China Securities Regulatory Commission; (4) Asset-liability ratio (*LEV*), which is the ratio of total liabilities to total assets of an enterprise; (5) Current ratio (*CR*), which is the ratio of current assets to total assets; (6) Return on equity (*ROE*), which is net profit after tax divided by net assets; (7) Independent Directors to Board (*DEDIRECTO*R) is the proportion of the company’s independent directors to the company’s board; (8) *AGE*, which is the time interval between the date of incorporation of the company and the statistical year.

### 3.2 Benchmark model: Two-way fixed effects model

This paper aims to study the relationship between corporate social responsibility investment and total factor productivity, explore how corporate social responsibility affects total factor productivity, and answer whether an enterprise can achieve a win-win between social value and commercial value. To test hypothesis H1: enterprises can improve total factor productivity by practicing the ESG concept, enhancing their long-term value, and realizing a win-win of social value and commercial value. Based on the theoretical models of Xu and Zhao [26], Ma, Gao [30], this paper constructs the following model to reflect the impact of ESG on TFP:

$$TFP=\alpha_{0}+\alpha_{1}ESG_{i,t-1}+\sum \alpha *control_{i,t-1}+\mu_{k}+\delta_{t}+\varepsilon_{i,t}$$ (2)

Among them, *ESG*_*i*, *t-1*_ represents the ESG performance of the i-th company in the t-1 year, *control*_*i*, *t-1*_ represents the control variables of the i-th company in the t-1 year, including firm size (*CORPSCALE*), firm nature (*NATURE*), industry (*INDUSTRY*), asset-liability ratio (*LEV*), current ratio (*CR*), return on equity (*ROE*), proportion of independent directors on the board of directors (*DEDIRECTOR*), and company age (*AGE*). However, *η*_*k*_ and *δ*_*t*_ represent the company’s individual and time-fixed effects respectively.

### 3.3 Data sources and processing

This paper selects A-share listed companies in the Chinese Shanghai and Shenzhen markets as research samples. Due to the limitation of ESG rating data, the sample period is selected as 2009–2021. All data are from the Wind Consulting Database. To ensure the accuracy of the data, the following screening and processing are carried out in this paper: (1) removing the missing samples of ESG data; (2) removing the samples of ST and ST*; (3) double-sided tail-shrinking for continuous variables, like *LEV*, *CR*, and *ROE* in 5% and 95% quantiles. Finally, the data of 1,631 listed companies in the Chinese Shanghai and Shenzhen markets from 2009 to 2021 are obtained.

Table 2 summarizes statistics of total factor productivity (*TFP*), the ESG performance (*ESG*), and related control variables of listed companies.

**Table 2:** Main variables descriptive statistics.

Variables	Sample	Mean	Std. Dev.	Minimum	Maximum
*TFP*	21060	17.717	1.480	5.972	23.868
*ESG*	21143	3.993	1.179	1	8
*CORPSCALE*	21203	3.764	0.529	1	4
*LEV*	21198	50.542	20.789	14.231	86.972
*CR*	21103	1.239	7.538	-14.356	16.097
*ROE*	20891	6.676	10.264	-20.579	25.092
*DEDIRECTOR*	21203	0.372	0.056	0.111	0.8
*NATURE*	21203	0.533	0.499	0	1
*INDUSTRY*	21203	5.326	3.716	1	18
*AGE*	21203	27.491	4.633	3	57

## 4. Basic results

### 4.1 The benchmark regression results

To test hypothesis 1, we first estimate the total factor productivity (*TFP*) of an enterprise by using the LP method as an explanatory variable, then perform a regression test according to model (2) to obtain the benchmark regression result Table 3. In Table 3, both the univariate regression results (column (1) and column (3)) and the regression results after adding the control variable (column (3) and column (4)) show that the coefficient of variable *ESG* is significantly positive at the level of 1%, indicating that the enterprise ESG does have a significant positive impact on total factor productivity. This conclusion has good robustness. It will not be changed due to whether to add the control variable or adopt different estimation methods. Chinese enterprises can improve total factor productivity by practicing the ESG concept and realizing a win-win of corporate social value and commercial value. There may be three main reasons: First, the promotion of ESG construction, such as fair remuneration system and employee stock ownership plan, is helpful to attract job-hunting talents to join, stimulate employees’ enthusiasm and initiative, improve employees’ work efficiency and promote total factor productivity; Second, the ESG concept helps to send positive signals to financial intermediaries such as banks and capital markets, improve stakeholders’ mastery of non-financial information of enterprises, and improve the accuracy of decisions made by financial institutions or investors, thus reducing their risk premium requirements and providing funding for technological innovation and equipment upgrades; Third, the reshaping of the business philosophy of the enterprise has enhanced the trust of various stakeholders in the enterprise, provided a relaxed environment for enterprise innovation investment, and helped to improve the willingness of enterprise innovation investment, thus promoting enterprise innovation and improving the total factor productivity of the enterprise.

**Table 3:** Regression results of benchmark model.

Variables	(1)	(2)	(3)	(4)
*ESG*	0.435*** (0.009)	0.271*** (0.007)	0.429*** (0.009)	0.266*** (0.007)
*Controls*	NO	YES	NO	YES
*Individual fixation effect*	NO	NO	YES	YES
*Year fixed effect*	NO	NO	YES	YES
*Constant*	15.98*** (0.037)	12.398*** (0.104)	15.368*** (0.052)	11.947*** (0.107)
*R-squared*	0.1196	0.4267	0.1463	0.4554
*Observes*	21000	20650	21000	20650

Note: *, * *, * * * are significant under the statistical levels of 10%, 5% and 1%, respectively. The brackets represent the robust standard error of clustering at the enterprise level. The regression in the table all controls the enterprise size, nature, industry, asset-liability ratio, current ratio, return on net assets, proportion of independent directors to the board of directors, enterprise age, and fixed effect. The following table is the same.

In the control variables, it can be demonstrated from Table 3 that the control variables, like *NATURE*, *LEV*, *CR*, *ROE*, and *DEDIRECTOR*, have significant positive effects on the total factor productivity of the enterprise. Variables like *INDUSTRY*, *CORPSCALE*, and *AGE*, have significant negative effects on total factor productivity. Variables like *CORPSCALE* and *DEDIRECTOR* have the most significant impact, consistent with our common sense. Compared with small and medium-sized enterprises and family enterprises, large and public enterprises tend to pay more attention to ESG construction, leading to a more perfect organizational structure and management system. As a result, financial institutions and investors have recognized these large and public enterprises, and staff cooperation efficiency and willingness to innovate are high, so they have a higher level of total factor productivity.

In a word, the benchmark results show that enterprises can improve total factor productivity, enhance their long-term value, and achieve a win-win situation of social value and commercial value by practicing the ESG concept. Hypothesis 1 has been verified.

### 4.2 The endogenous problem: Instrumental variable method

To alleviate the endogenous problems caused by the omission of significant explanatory variables and reverse causality, exploring the genuine causal relationship between ESG performance and total factor productivity, this paper will further analyze using the instrumental variable method. Previous literature has often used industry or regional ESG levels as a tool variable for corporate ESG [31]. Although such instrument variables easily meet the correlation requirements, there is a problem that the exclusivity principle cannot be satisfied significantly when the samples are affected by the common fixed effect. The ESG level of the industry or region may not affect the total factor productivity of the enterprise through the enterprise ESG [32]. Therefore, this paper takes the quantity (*FQ*) and market value (*FV*) held by the "Broad ESG" fund as the two instrumental variables of ESG.

The shareholding information of the "Broad ESG" fund as a tool variable is mainly based on the following considerations: Firstly, as an important institutional investor, the fund will influence corporate decision-making through "voting with one’s feet." The investment philosophy of ESG funds will affect the operation and management of listed companies, thus improving the performance of ESG [21], thus meeting the principle of relevance of tool variables. Secondly, as the "Broad ESG" fund seldom directly interferes with the company’s business decisions, the shareholding information only relates to the fund company itself, which has no direct connection with the listed company’s total factor productivity, thus satisfying the exclusivity principle of instrumental variables. To sum up, the shareholding information of the " Broad ESG" fund as a tool variable satisfies the requirements of both "relevance" and "exclusivity".

As ESG affects total factor productivity with a certain lag, the two instrument variables, the quantity of holding funds (*FQ*) and market value of holding funds (*FV*), are both taken as lag, and the logarithm of the market value of holding funds (*lnFV*) is used to alleviate the heteroscedasticity problem. Then, based on the two-stage least square method, Table 4 summarizes the regression results of the instrumental variable method. Column (1) in Table 4 shows the regression results of the first stage of the instrumental variable method. The results show that both instrumental variables are significantly positive, indicating that there is a significant positive relationship between the two instrumental variables and endogenous variables. The greater the quantity of " Broad ESG " funds held (*FQ*) or the larger the market value held (*FV*), the more institutional investors will participate in the governance of listed companies and promote the improvement of the ESG of listed companies. Table 4 Test results also show that there are no weak tool variables and over-identification problems. In particular, the partial *R*^*2*^ of the first-stage regression of the instrumental variable method is as high as 5.53%, which proves that the instrumental variable has strong marginal explanatory power. Column (2) in Table 4 shows the second-stage regression results, which show that the ESG coefficient is significantly positive, and the total factor productivity of the enterprise will increase by 1.809 basis points for every level of ESG increase. To sum up, after controlling endogeneity with the instrumental variable method, enterprises can still improve total factor productivity by practicing the ESG concept, enhancing their long-term value, and realizing a win-win of social value and commercial value.

**Table 4:** Two-stage least squares regression results of instrumental variables.

Variables	(1)	(2)
	The First Stage: *ESG*	The Second Stage: *TFP*
*ESG*		1.809*** (0.106)
*FQ*	0.001*** (0.0001)	
*lnFV*	0.063*** (0.0056)	
*Controls*	YES	YES
*Individual fixation effect*	YES	YES
*Year fixed effect*	YES	YES
*Weak instrumental variable test*	364.629 [P Value:0.000]	
*Over-identification test*	0.966 [P Value:0.326]	
*Partial R-squared*	0.0553	
*Observes*	18641	18641

### 4.3 The robustness test

(1) Considering the impact of the core explanatory variable measure. The ESG ratings were assigned above. To test the effect of the assignment method on the regression results, we re-use two assignment methods to measure the ESG of the enterprise.

The first assignment method is a piecewise assignment. From the descriptive statistics of the main variables in Table 2, it can be found that the average value of the ESG rating data is 3.99 (level B). Therefore, this paper changes the assignment method of ESG rating data, assigning 1 above level B and 0 for others. As can be seen from Table 5, enterprises can improve total factor productivity by practicing the ESG concept and realizing a win-win of social value and commercial value. Therefore, the result of re-estimating the core explanatory variables by using the method of piecewise assignment is consistent with the previous conclusion.

**Table 5:** Regression results of re-estimation after changing ESG rating method to piecewise assignment.

Variables	(1)	(2)	(3)	(4)
*ESG*	0.816*** (0.019)	0.506*** (0.016)	0.782*** (0.019)	0.467*** (0.016)
*Controls*	NO	YES	NO	YES
*Individual fixation effect*	NO	NO	YES	YES
*Year fixed effect*	NO	NO	YES	YES
*Observes*	21060	20673	21060	20673
*R-squared*	0.0725	0.4044	0.0944	0.4270

Next, we use the second assignment method, virtual variable assignment, that is, each rating generates a 0–1 virtual variable and adds it to the model. As Huazheng ESG has 9 grades, model (2) introduces 8 virtual variables of ESG to replace the core explanatory variable ESG. The virtual variables with grades from C to AA are ESG_ 1 to ESG_ 8, and then regress. As can be seen from Table 6, a low ESG rating (i.e. ESG_1 to ESG_2 with a rating below "CC") will hinder the improvement of total factor productivity, and the lower the ESG rating, the stronger the hindrance; A high ESG rating (i.e. ESG_ 4 to ESG_ 8 with a rating above "B") will promote the improvement of total factor productivity, and the higher the ESG rating, the stronger the promotion effect. Therefore, enterprises can improve total factor productivity by practicing the ESG concept and realizing a win-win of social value and commercial value. Therefore, the result of re-estimating the core explanatory variables by using the method of virtual variable assignment is consistent with the previous conclusion.

**Table 6:** Regression results of re-estimation after changing ESG rating method to virtual variable assignment.

Variables	(1)	(2)	(3)	(4)
*ESG_1*	−0.531*** (0.127)	−0.297** (0.138)	−0.905*** (0.121)	−0.477*** (0.12)
*ESG_2*	−0.388*** (0.119)	−0.279** (0.133)	−0.709*** (0.111)	−0.404*** (0.115)
*ESG_3*	0.118 (0.113)	0.028 (0.129)	−0.171* (0.105)	−0.05 (0.11)
*ESG_4*	0.483*** (0.112)	0.206* (0.128)	0.21** (0.103)	0.15** (0.109)
*ESG_5*	0.926*** (0.112)	0.509*** (0.128)	0.619*** (0.104)	0.416*** (0.109)
*ESG_6*	1.422*** (0.116)	0.888*** (0.13)	1.051*** (0.108)	0.726*** (0.111)
*ESG_7*	1.757*** (0.754)	1.176*** (0.149)	1.296*** (0.151)	0.92*** (0.134)
*ESG_8*	2.687*** (0.111)	1.759*** (0.128)	2.297*** (0.109)	1.479*** (0.113)
*Controls*	NO	YES	NO	YES
*Individual fixation effect*	NO	NO	YES	YES
*Year fixed effect*	NO	NO	YES	YES
*Observes*	21060	20673	21060	20673
*R-squared*	0.1163	0.4188	0.1395	0.4427

(2) Considering the impact of the measurement method of the explained variable. At present, the accounting methods of total factor productivity (*TFP*) of enterprises include the LP method, the OP method, and ACF method, etc. However, due to the existence of labor protection law in China, the labor adjustment cost problem of enterprises in China is not serious, and the premise assumption of the ACF method is not in conformity with the actual situation in China. Therefore, this paper only uses the OP method to re-estimate the total factor productivity (*TFP*) of listed companies and test the impact of the change in the measurement method of the explained variable on the results. The agency variable in the OP method uses "cash paid for the purchase of fixed assets, intangible assets, and other long-term assets", while the "exit variable" of a listed company is determined based on whether the listed Company is ST, *ST and PT within the sample range and whether it is delisted. As can be seen from Table 7, the results of the re-estimation of the explained variables by using different measurement methods are consistent with the previous conclusions.

**Table 7:** Regression results of re-estimation after changing the measurement method of TFP.

Variables	(1)	(2)	(3)	(4)
*ESG*	0.414*** (0.009)	0.307*** (0.008)	0.408*** (0.009)	0.304*** (0.008)
*Controls*	NO	YES	NO	YES
*Individual fixation effect*	NO	NO	YES	YES
*Year fixed effect*	NO	NO	YES	YES
*Observes*	21000	20940	21000	20940
*R-squared*	0.1149	0.2775	0.1385	0.2972

(3) Considering the effect of time series correlation of total factor productivity. As the total factor productivity of enterprises has a strong correlation in time, we re-estimate the impact of ESG level on total factor productivity after adding the lag term (TFP_-1) of TFP to test the robustness of the conclusion. As can be seen from Table 8, the results of the re-estimation after considering the effect of the time-series correlation of total factor productivity are consistent with the previous conclusions.

**Table 8:** Regression results of re-estimation after addition of the TFP lag term.

Variables	(1)	(2)	(3)	(4)
*ESG*	0.027*** (0.004)	0.019*** (0.003)	0.028*** (0.004)	0.021*** (0.003)
*TFP_-1*	0.942*** (0.006)	0.912*** (0.006)	0.944*** (0.006)	0.913*** (0.006)
*Controls*	NO	YES	NO	YES
*Individual fixation effect*	NO	NO	YES	YES
*Year fixed effect*	NO	NO	YES	YES
*Observes*	19370	19129	19370	19129
*R-squared*	0.8883	0.9037	0.8902	0.9055s

## 5. Mechanism analysis

Through the above analysis, hypothesis 1 has been verified, and enterprises can indeed improve total factor productivity, enhance their long-term value, and realize a win-win of social value and commercial value by practicing the ESG concept. Next, this paper will further study the mechanism of ESG’s impact on total factor productivity from the perspectives of employee efficiency, capital constraint, and innovation investment. Referring to the mechanism analysis framework of Xu and Zhao [26], Chen, Ma [29], the following intermediate mechanism analysis model is constructed:

$$MV_{\mathrm{it}}=\beta_{0}+\beta_{1}ESG_{i,t-1}+\sum \beta *control_{i,t-1}+\mu_{k}+\delta_{t}+\varepsilon_{i,t}$$ (3)

$$TFP_{it}=\gamma_{0}+\gamma_{1}MV_{it}+\sum \gamma *control_{i,t‐1}+\mu_{k}^{'}+\delta_{t}^{'}+\varepsilon_{i,t}^{'}$$ (4)

Among them, *MV*_*it*_ represents the value of the intermediary variable in the t period of the i-listed company, and other variables are consistent with the model mentioned above. Model (3) is used to test the influence of enterprise ESG on the intermediary variable (*MV*), and model (4) is used to test the influence of the intermediary variable (*MV*) on the total factor productivity (*TFP*).

### 5.1 Staff efficiency mechanism

As the core element of enterprise productivity development, the labor force plays an important role. How to motivate the staff’s initiative and improve the staff’s efficiency has become the key to improving total factor productivity. The implementation of the ESG concept extends the "maximization of shareholders’ interests" to stakeholders, sending a positive signal to society and helping to attract talented job seekers. At the same time, ESG construction means paying attention to employee benefits, promoting the alignment of employees’ and enterprises’ interests through means such as employee shareholding plans and salary incentives, improving employees’ initiative and enthusiasm, enhancing employees’ production cooperation efficiency, and thus promoting total factor productivity. Labor productivity refers to the ratio of the expected labor results created by workers within a certain period to the corresponding labor consumption [33]. It is the main method to verify the efficiency of unit labor production and service. Referring to the method of Delmas and Pekovic [34], labor productivity (*LP*) is obtained by dividing the output (measured by "operating income") by the total number of employees, which is used as the intermediate variable (*MV*) of ESG that affects the total factor productivity of the enterprise, and is substituted into model (3) and model (4) to test the intermediate effect of employee efficiency.

According to the regression results in Table 9, column (1) is the regression result of the model (3), which is used to test the impact of ESG on employee labor efficiency (*LP*). The coefficient of ESG is significantly positive, indicating that the higher the ESG level of an enterprise, the higher the employee labor efficiency. Column (2) is the regression result of the model (4), and the coefficient of variable *LP* is still significantly positive, indicating that employee labor efficiency can indeed promote enterprise total factor productivity. To sum up, enterprises can indeed improve staff labor efficiency by practicing the ESG concept, thus improving total factor productivity. Staff efficiency is one of the potential mechanisms for ESG to promote total factor productivity. Hypothesis 2 holds.

**Table 9:** Estimated results of testing staff efficiency as a potential mechanism.

Variables	(1)	(2)
	Model (3): *LP*	Model (4): *TFP*
*ESG*	0.106*** (0.045)	
*LP*		0.03*** (0.01)
*Controls*	YES	YES
*Individual fixation effect*	YES	YES
*Year fixed effect*	YES	YES
*Observes*	20755	20667
*R-squared*	0.0265	0.425

### 5.2 Capital restraint mechanism

The constraint of external financing constraint on enterprise development has been generally verified [22]. The implementation of the ESG concept by enterprises can help to improve the financing difficulties caused by information asymmetry, improve investors’ and financial institutions’ grasp of non-financial information of enterprises, and the accuracy of investment decisions. So that enterprises can use more cheap funds for technological innovation and equipment upgrading, improving the total factor productivity of enterprises, and achieving high-quality development. In the following, we will examine the potential mechanism of ESG’s impact on total factor productivity from the perspectives of financing cost and financing constraint.

Regarding the financing cost (*DebtCost*), referring to the practice of El Ghoul, Guedhami [3], Goss and Roberts [4], the "ratio of interest payable to total liabilities" of listed companies is used to measure the cost of financing (*DebtCost*) of listed companies in the current year. Regarding the financing constraint (*SA*), the methods to measure corporate financing constraints are the KZ index, WW index, and SA index. The first two indexes contain financial indexes such as cash flow and leverage that are easy to affect each other with financing constraints. Therefore, to avoid endogenous interference, this paper refers to Yu, Wu [22], and uses the SA index to measure the financing constraint of listed companies:

$$SA_{t}=-0.737*Size_{t}+0.043*Size_{t}^{2}-0.04*Age_{t}$$ (5)

In terms of testing the financing cost (*DebtCost*) mechanism, column (1) in Table 10 is the regression result of model (3), and the ESG coefficient is significantly negative, indicating that the higher the ESG level of an enterprise, the more helpful it is to reduce its financing cost; While column (2) is the regression result of model (4), the coefficient of the intermediate variable (*MV*) is still significantly negative, indicating that reducing the financing cost can promote the total factor productivity of enterprises. To sum up, it is confirmed that ESG strives to reduce financing costs and improve total factor productivity. Therefore, reducing financing costs is indeed one of ESG’s potential mechanisms to promote total factor productivity.

**Table 10:** Estimated results of testing the financing cost and financing constraint as a potential mechanism.

Variables	Financing Cost(*DebtCost*)		Financing Constraint(*SA*)	
	(1)	(2)	(3)	(4)
	Model (3)*MV*	Model (4)*TFP*	Model (3)*MV*	Model (4)*TFP*
*ESG*	-0.001*** (0.0002)		-0.018*** (0.0009)	
*MV*		-10.509*** (0.746)		-3.137*** (0.073)
*Controls*	YES	YES	YES	YES
*Individual fixation effect*	YES	YES	YES	YES
*Year fixed effect*	YES	YES	YES	YES
*R-squared*	0.0556	0.3961	0.7194	0.478
*Observes*	13467	13433	20768	20673

In terms of testing the financing constraint (*SA*) mechanism, column (3) in Table 10 is the regression result of model (3), and the coefficient of ESG is significantly negative, indicating that the higher the ESG level of an enterprise, the more helpful it is to ease its financing constraint; Column (4) is the regression result of model (4), and the coefficient of the intermediate variable (*MV*) is significantly negative, indicating that easing the financing constraint (*SA*) can promote total factor productivity. In conclusion, it is confirmed that ESG efforts can ease financing constraints and improve total factor productivity. Therefore, easing financing constraints is indeed one of ESG’s potential mechanisms to promote total factor productivity. To sum up, enterprises can reduce financing costs and ease financing constraints by implementing the ESG concept, thus improving total factor productivity. Hypothesis 3 holds.

### 5.3 Innovative investment mechanism

The implementation of the ESG can help alleviate the principal-agent problem between owners and managers, promoting the Innovation investment of enterprises, and thus improving the total factor productivity. Due to the large uncertainty of innovation investment, both managers and stakeholders are more inclined to short-term projects with stable cash flows, so the enterprise innovation investment is insufficient. The implementation of the ESG concept can send a positive signal to the outside world that enterprises are fulfilling their social responsibilities, increase the trust of stakeholders such as investors, and provide a relaxed environment for enterprise innovation, thus increasing the willingness of enterprises to invest in innovation. At the same time, the performance appraisal incorporating the ESG concept also urges professional managers to pay more attention to the long-term development of the enterprise and promote the innovation investment of the enterprise, thus improving the total factor productivity.

This paper uses the methods of Sun, Zhang [35] to measure the level of innovation investment (*RD*) by using the logarithmic per capita R&D expenditure as an intermediate variable (*MV*) that ESG affects the total factor productivity of enterprises, and uses model (3) and model (4) to test the intermediate effect of innovation investment. According to the regression results in Table 11, column (1) is the regression result of the model (3), which is used to test the impact of ESG on innovation investment level (*RD*). The coefficient of variable *ESG* is significantly positive, indicating that the higher the ESG level of an enterprise, the higher the innovation investment level. Column (2) is the regression result of the model (4), and the coefficient of variable *RD* is still significantly positive, indicating that Innovation investment level (*RD*) can indeed promote enterprise total factor productivity. In conclusion, it has been proved that enterprises can indeed improve the level of Innovation investment by practicing the ESG concept, thus improving total factor productivity. Innovation investment is one of the potential mechanisms for ESG to promote total factor productivity. Hypothesis 4 holds.

**Table 11:** Estimated results of testing innovation investment as a potential mechanism.

Variables	(1)	(2)
	Model (3): *RD*	Model (4): *TFP*
*ESG*	0.087*** (0.012)	
*RD*		0.046*** (0.006)
*Controls*	YES	YES
*Individual fixation effect*	YES	YES
*Year fixed effect*	YES	YES
*Observes*	14428	14378
*R-squared*	0.1283	0.4015

## 6. Expansion analysis

### 6.1 ESG acceleration effect

Through the above theoretical analysis and empirical test, we confirmed that enterprises can improve total factor productivity, enhance their long-term value, and achieve a win-win of social value and commercial value by practicing the ESG concept, and studied its impact mechanism in detail. Through practical observation, we also found that with the improvement of the relevant ESG disclosure, supervision, and other institutional mechanisms in China, ESG in China ushered in accelerated development. In 2022, 1,758 A-share listed companies disclosed ESG-related reports, while in 2019, the number was only 371. The scale of China’s " Broad ESG" fund has also increased from 29.44 billion yuan in 2013 to 135.498 billion yuan in 2022, an increase of nearly 46 times. More and more resources are inclined towards ESG construction. Does ESG also have an accelerating effect on the promotion of total factor productivity of enterprises? To verify this problem, based on the previous model (2), this paper refers to the methods of Yu, Guo [36], adds the quadratic term of ESG, and constructs the following model:

$$TFP=\nu_{0}+\nu_{1}ESG_{i,t-1}+\nu_{2}ESG_{i,t-1}^{2}+\sum \nu *control_{i,t-1}+\mu_{k}+\delta_{t}+\varepsilon_{i,t}$$ (6)

Among them, *ESG*_*i*,*t-1*_, *ESG*^*2*^_*i*,*t-1*_ are the primary and secondary terms of ESG respectively, and are key explanatory variables, representing the ESG performance of th-i company in the t-1 year. *control*_*i*,*t-1*_ indicates all control variables in year t-1 of the company; *μ*_*k*_ and *δ*_*t*_ are firm individual and time fixed effects respectively.

To verify whether enterprise ESG has an accelerating effect on total factor productivity, we tested the nonlinear relationship between ESG and total factor productivity according to model (6) and got the regression result of the model (6) in Table 12. The results show that the regression coefficients of both primary and secondary terms of enterprise ESG are significantly positive. Once again, the establishment of Hypothesis 1 is verified. The enterprise can indeed improve the total factor productivity by practicing the ESG concept and realizing a win-win of social value and commercial value. In addition, the quadratic formula also shows that the relationship between enterprise ESG and total factor productivity is the right half of a parabola with an upward opening, namely a curve inclined to the upper right, which proves that the promotion effect of enterprise ESG on enterprise total factor productivity is also accelerating.

**Table 12:** Regression results of the model after adding ESG quadratic terms.

Variables	(1)	(2)	(3)	(4)
*ESG*	0.264*** (0.042)	0.162*** (0.037)	0.395*** (0.044)	0.178*** (0.034)
*ESG* ^*2*^	0.022*** (0.005)	0.023*** (0.005)	0.005** (0.004)	0.011*** (0.004)
*Controls*	NO	YES	NO	YES
*Individual fixation effect*	NO	NO	YES	YES
*Year fixed effect*	NO	NO	YES	YES
*R-squared*	0.1204	0.3552	0.1463	0.4556
*Observes*	21000	20650	21000	20650

This conclusion is closely related to the reality in China. Compared with the developed countries in Europe, the development of social responsibility investment in China is relatively late and is in the early stage of development. With the continuous improvement of relevant systems and mechanisms, the development of social responsibility investment has been accelerated. Relevant funds in the capital market have been increasing year by year, and resources such as policies and funds have been continuously injected. Therefore, compared with the international market, ESG in China has an accelerating effect on total factor productivity. In addition, as the relationship between ESG and total factor productivity is the right half of a parabola, ESG’s contribution to total factor productivity may have a certain limit.

### 6.2 Heterogeneity test

To further investigate the differential impact of ESG performance of enterprises with different ownership and sizes on total factor productivity, this paper divides the sample enterprises into large enterprises and medium-small enterprises, state-owned enterprises and non-state-owned enterprises according to ownership and size types, and then performs different sample regressions. Finally, based on the seemingly unrelated model SUR, it performs the inter-group regression coefficient differential test to obtain the estimation results in Table 13.

**Table 13:** Regression results of the heterogeneity test.

Variables	Total factor productivity(*TFP*)			
	Classification by ownership		Classification by sizes	
	(1)	(2)	(3)	(4)
	State-owned Enterprises	Non-state-owned Enterprises	Large Enterprises	Medium-small Enterprises
*ESG*	0.264*** (0.01)	0.237*** (0.01)	0.241*** (0.008)	0.247*** (0.018)
*Controls*	YES	YES	YES	YES
*Individual fixation effect*	YES	YES	YES	YES
*Year fixed effect*	YES	YES	YES	YES
*Observes*	20650	20650	20650	20650
*Coefficient difference test between groups*	χ^2^(1) = 3.63 P>χ^2^ = 0.0567		χ^2^(1) = 0.12 P>χ^2^ = 0.73	

Column (1) and column (2) in Table 13 show the differentiated impact of ESG levels of state-owned enterprises and non-state-owned enterprises. The ESG coefficients are significantly positive, indicating that both state-owned enterprises and non-state-owned enterprises can improve total factor productivity through ESG efforts to achieve a win-win situation of social value and commercial value. However, the difference test of regression coefficients between groups shows that the ESG coefficients are significantly different at the level of 10%, and the ESG coefficients of state-owned enterprises are significantly larger, indicating that compared with non-state-owned enterprises, the state-owned enterprises strive to improve total factor productivity better through ESG. Column (3) and column (4) in Table 13 show the differentiation effect of ESG levels of large enterprises and medium-small enterprises. Their ESG coefficients are significantly positive and the coefficient difference is small, and the coefficient difference test between groups also shows that there is no significant difference between the two groups. Therefore, large enterprises and medium-small enterprises can improve total factor productivity by practicing the ESG concept, and the effect is not significantly different. To sum up, large enterprises and medium-small enterprises, state-owned enterprises and non-state-owned enterprises, can improve total factor productivity through the implementation of the ESG, but the effect of state-owned enterprises is better than that of non-state-owned enterprises.

## 7. Main conclusions

With sustainable problems such as climate change, energy shortage, and the gap between the rich and the poor becoming increasingly prominent, the related social norms and policy regulations are becoming more and more strict, which drives enterprises to continuously increase relevant investment, to expect to achieve a win-win situation of corporate social value and commercial value. one of the crucial controversies is whether a large amount of resources invested in ESG construction will sacrifice corporate economic efficiency. However, existing literature mostly focuses on exploring the impact of ESG on enterprise commercial value, and there is no clear answer to the relationship between ESG and the quality of enterprise development (production efficiency). This article clarifies the impact and mechanism of corporate ESG on total factor productivity through empirical analysis, filling the research gap mentioned above and helping to better optimize the relationship between corporate social responsibility and total factor productivity, achieving a win-win of corporate business value and social value. This study found that Chinese enterprises can indeed improve staff labor efficiency, reduce financing costs, ease financing constraints, and increase innovation investment through ESG construction, to effectively enhance total factor productivity, and achieve a win-win between social value and commercial value. And the effect is especially significant for state-owned enterprises. In addition, the promotion of ESG construction on enterprise total factor productivity also shows certain acceleration characteristics. We control the potential endogeneity problem by utilizing the instrumental variable method. To further test the robustness of conclusions, we replace different assignments and different measures, and add time lag terms, etc. On the whole, the research in this paper indicates that there is an obvious "social responsibility dividend" in the practice of the ESG concept in China. It indicates that Chinese enterprises need to pay more attention to the social benefits of corporate behavior and activities through ESG construction, seize the opportunity to obtain social responsibility dividends, and realize the win-win of social value and commercial value.

With the complex international environment, the triple pressures of shrinking demand, supply shocks, and weakening expectations are constantly emerging, economic development is facing an unprecedented severe situation. Under this background, this paper discusses the relationship and transmission mechanism between corporate social responsibility and total factor productivity through empirical research. It theoretically clarifies the urgency for enterprises to seize the opportunity of the times to promote the development of ESG and helps to optimize and formulate an effective way for sustainable development.

To sum up, the research in this paper has the following implications for promoting sustainable development: Firstly, in line with the requirements of sustainable development, enterprises should grab the "social responsibility dividend", reshape the corporate business philosophy, carry out ESG construction, and improve total factor productivity. Secondly, enterprises should attach great importance to the interests of employees, promote the interests of employees and enterprises to be consistent through employee stock ownership plans, salary incentives and other means, thus stimulating the enthusiasm and creativity of employees, and improving the efficiency of production cooperation. Thirdly, corporates should actively disclose ESG information, improve internal and external information asymmetry, and improve the decision-making accuracy of financial institutions and investors, to ease financing constraints, reduce financing costs, and provide financial support for technological innovation and equipment upgrades. Fourthly, corporates should give full play to the guiding role of ESG. Through incorporating the ESG concept into performance evaluations, enterprise managers are urged to pay more attention to the long-term development of the business, and promote Innovation investment, thus raising the level of technology innovation to improve total factor productivity.

## Supporting information

S1 Data (XLSX)
